# Microscope-Integrated Optical Coherence Tomography-Guided Rapid Resolution of Acute Hydrops

**DOI:** 10.7759/cureus.77049

**Published:** 2025-01-06

**Authors:** Deepak Kumar, Aafreen Bari, Vishal Jhanji, Namrata Sharma, Tushar Agarwal

**Affiliations:** 1 Ophthalmology, All India Institute of Medical Sciences, New Delhi, IND; 2 Cornea, Cataract, and External Disease Services, University of Pittsburgh School of Medicine, Pittsburgh, USA

**Keywords:** acute hydrops, intracameral c3f8, keratoconus, mi-oct, rapid resolution

## Abstract

An 18-year-old boy presented with blurred visual acuity in the left eye for three days associated with mild pain and photophobia. On examination, he had diffuse corneal stromal edema. Anterior segment optical coherence tomography (AS-OCT) revealed stromal edema with a large hypodense fluid pocket in the posterior stroma. A diagnosis of acute hydrops with an intrastromal cleft in the left eye was made, and he was planned for intracameral gas injection. Intraoperatively, microscope-integrated OCT (Mi-OCT)-guided intracameral isoexpansile sulphur hexafluoride(SF6) was injected ensuring an 80% fill of the anterior chamber. Mi-OCT-assisted venting incision with drainage of stromal fluid clefts was done. On the first postoperative day, the fluid pocket and stromal edema resolved completely. At six weeks' follow-up, the central cornea remained clear with the formation of a paracentral scar. Thus, intracameral gas combined with stromal cleft drainage appears to be a highly effective management option for acute hydrops with a single large stromal fluid cleft. Intraoperative OCT (Mi-OCT) can guide real-time drainage of intrastromal fluid clefts, facilitating the closure of focal Descemet's membrane (DM) defects and enabling early visual rehabilitation, as demonstrated in this case as early as one-day postprocedure.

## Introduction

Corneal hydrops is an acute condition characterized by the sudden onset of corneal stromal edema, usually more severe in the paracentral cornea, noted in slit-lamp examinations as a protruding, opaque area on the cornea, which is caused by a break in Descemet's membrane that allows aqueous humor to leak into the corneal stroma. Sometimes pockets of fluid of different sizes (intrastromal clefts) may form [1-3]. Other associated clinical signs include overlying epithelial edema and conjunctival congestion. It is typically associated with advanced keratoconus; however, it has also been reported in other corneal ectasias like pellucid marginal degeneration, Terrien's marginal degeneration, keratoglobus, and post-laser-assisted in situ keratomileusis ectasia [4]. Acute hydrops occurs in approximately 1.4% to 3% of eyes with keratoconus primarily in the younger age group [4-6]. Eye rubbing appears to be one of the most important risk factors in the development of acute hydrops, which may be secondary to vernal keratoconjunctivitis (VKC), atopic kerato-conjunctivitis, and uncorrected refractive error [6,7]. The most common presentation of acute hydrops is a sudden severe reduction in visual acuity, which is associated with intense photophobia and watering. Ultrasound biomicroscopy and anterior segment optical coherence tomography (AS-OCT) are valuable imaging modalities for assessing the depth, location, type, and extent of edema. They also help characterize intrastromal clefts and determine the location, size, and orientation of Descemet's membrane tears [2,3]. Acute hydrops can be treated with conservative treatment (hypertonic saline eye drops, intraocular pressure lowering agent, cycloplegic, antibiotic, steroid) or surgical intervention like intracameral injection of air/gas, compressive suturing along with gas injection, penetrating keratoplasty, and amniotic membrane transplantation (AMT) with cauterization. The resolution with medical management typically takes several weeks to a few months, but the presence of intrastromal clefts can prolong this process, sometimes extending beyond six months [3,8-10]. We aim to report a case of acute hydrops that resolved within 24 hours of management and study the preoperative and intraoperative factors responsible for the same.

## Case presentation

**Figure 1:**
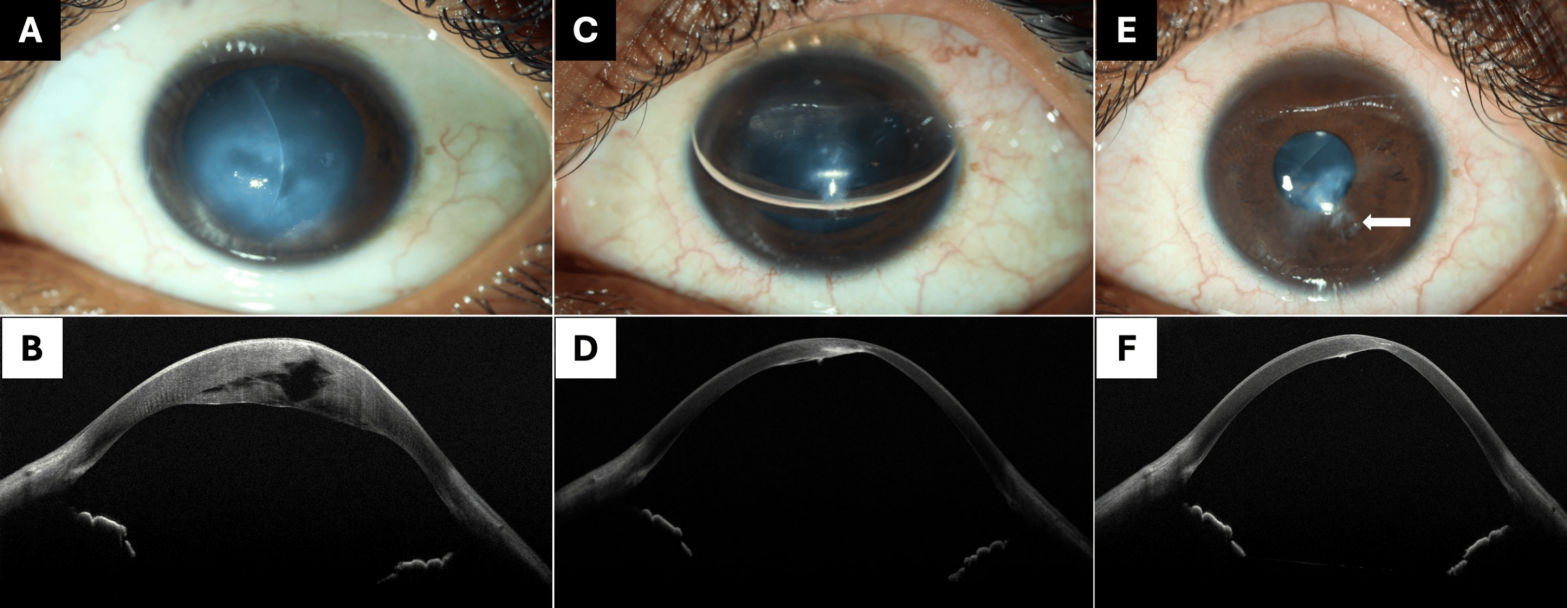
Clinical image and AS-OCT picture AS-OCT: anterior segment optical coherence tomography (A) Clinical image of the case of acute hydrops at presentation. (B) AS-OCT at presentation showing a single large hypodense intrastromal fluid pocket. (C) Clinical image at the first postoperative day. (D) AS-OCT at the first postoperative day showing compact stroma. (E) Clinical image at one-month follow-up with scar formation; the arrow shows a scar at the site of venting incision. (F) AS-OCT at one-month follow-up shows hyporeflective posterior corneal scar with the absence of stromal edema

An eighteen-year-old boy presented to the eye casualty with intense photophobia, watering, blurred vision, and pain in the left eye for three days. He was a known case of keratoconus with allergic kerato-conjunctivitis affecting both eyes. He had a history of vigorous eye rubbing. There was no history of trauma, contact lens wear, or any recent surgery. He did not have any known systemic illness. The visual acuity at the presentation was finger counting at one meter and hand movements close to the face in the right and left eyes, respectively. The intraocular pressures were 14 mm Hg in both eyes. On clinical examination, he had diffuse central corneal edema in the left eye (Figure 1A). The right eye had corneal steepening with a central corneal scar. Both eyes showed the presence of palpebral conjunctival papillae. AS-OCT of the left eye revealed stromal edema with a large stromal fluid cleft with no obvious break in DM (Figure 1B). A diagnosis of both eyes advanced keratoconus associated with VKC, right eye healed hydrops and left eye acute corneal hydrops was made.

**Figure 2:**
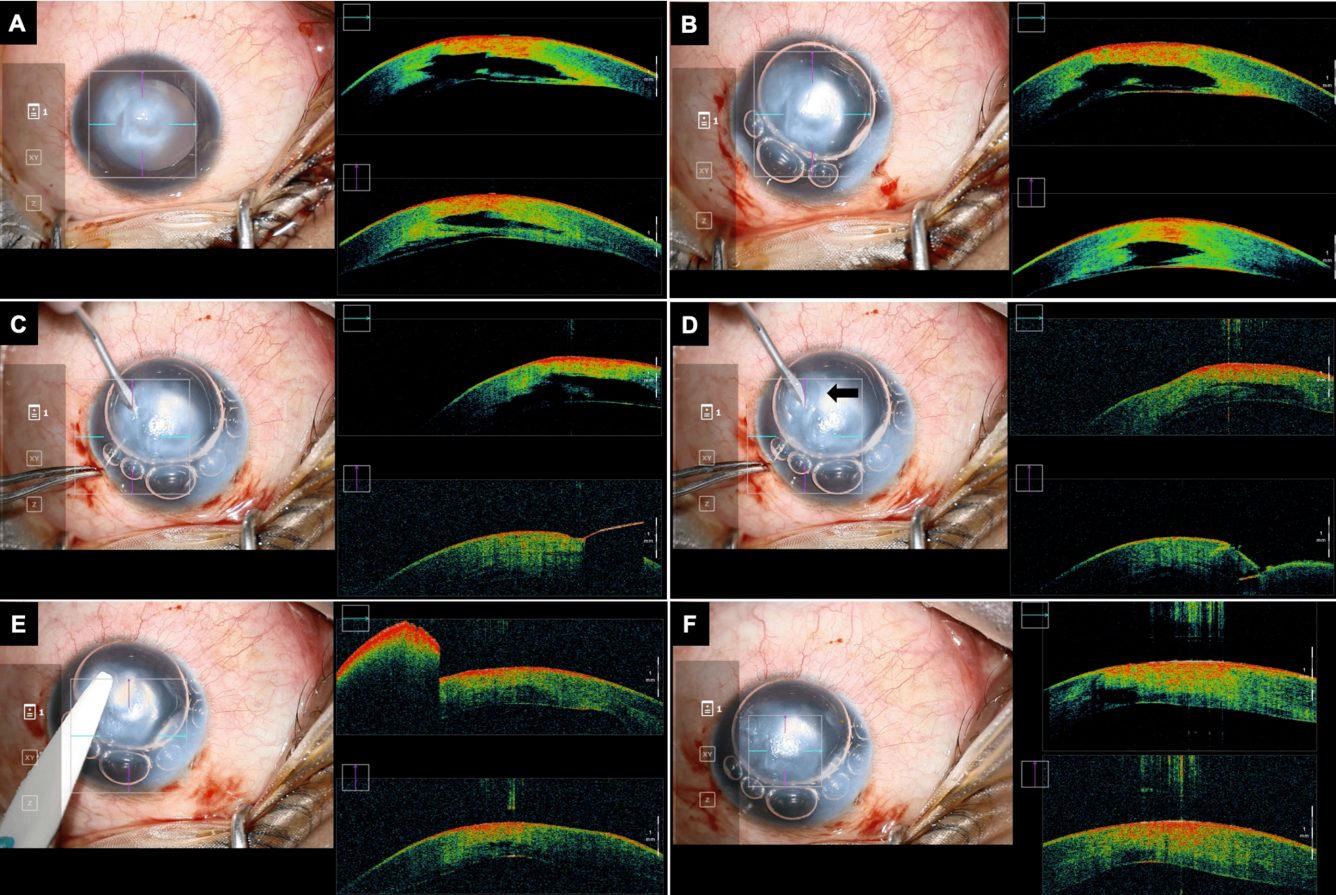
Surgical step and Mi-OCT images at various surgical steps Mi-OCT: microscope-integrated optical coherence tomography (A) Localisation of intrastromal cleft. (B) Injection of intracameral gas. (C) Inferotemporal venting incision toward intrastromal fluid cleft. (D) Fluid expression (arrow) after creation of venting incision. (E) Polyvinyl acetal sponges used for soakage of egressed fluid. (F) At the end of the procedure, hypodense fluid cleft has minimized in volume

The patient was planned for left eye intracameral gas under peribulbar anesthesia. Intraoperatively, microscope-integrated OCT (Mi-OCT) helped to localize the stromal fluid cleft (Figure 2A). A single limbal microvitreoretinal incision was made. A total of 20% sulfur hexafluoride injection was prepared and injected intracamerally. After 80% coverage of the anterior chamber, the incision was hydrated (Figure 2B). A venting incision was made using an angled microvitreoretinal blade from the inferotemporal quadrant of the cornea (Figure 2C). The entry and direction of the blade were directed toward the stromal fluid pocket which was observed as hyporeflective foci on Mi-OCT. On reaching the targeted area, the blade was retracted back-creating a tract for fluid egression (Figure 2D). It was dried with polyvinyl acetal sponges (Merocel, Beaver-Visitec, MA) (Figure 2E). Mi-OCT showed decreased intrastromal fluid cleft volume with a relatively compact stroma (Figure 2F). Postoperatively, he was started with topical antibiotics (moxifloxacin 0.5% three times a day), corticosteroids (prednisolone phosphate 1% four times a day), and cycloplegics (homatropine 2% four times a day). He was advised to maintain a supine position for the day.

On postoperative day one, the corneal clarity improved with the resolution of stromal edema. There was an air bubble occupying three-fourths of the anterior chamber (Figure 1C). On AS-OCT, the stromal lamellae were compact, and the stromal fluid pocket had nearly resolved (Figure 1D). At the one-month follow-up, the cornea remained clear with the formation of a paracentral corneal scar (arrow, Figure 1E). The uncorrected visual acuity improved to log MAR 0.6. On AS-OCT, the cornea was compact with no fluid cleft and posterior corneal scar (Figure 1F).

## Discussion

Acute hydrops is usually associated with keratoconus and a history of eye rubbing. Mechanical trauma from eye rubbing is a known risk factor for developing acute hydrops in patients with keratoconus. The average resolution time ranges from several weeks to a few months and may extend further in cases involving stromal clefts [3,5,9,10]. The management of acute hydrops is either medical or surgical. The latter is preferred for early resolution of symptoms and to prevent corneal neovascularization. The surgical treatment involves the use of intracameral gas or air, venting incisions, and compression sutures. The comparison between full-thickness and partial-thickness compression sutures has also been studied.

This case is unique due to the rapid and complete resolution of acute hydrops on the first postoperative day. The likely causes are a combination of anatomical and intraoperative factors. The presence of a unique large intrastromal cleft made it suitable for creating a single venting incision. Mi-OCT guided the precise execution of the surgical steps. It helped to localize the fluid cleft in the posterior stroma of an otherwise hazy cornea. It also assisted in the creation of the venting incision, allowing stromal fluid to drain from the pocket. Intracameral air provided counter-support during the incision and helped seal any micropunctures in the DM that were not detectable on clinical examination or AS-OCT. Additionally, it helped maintain the dehydrated state of the corneal stroma by refraining contact with aqueous humor intraoperatively as well as during the early postoperative period.

The use of intracameral gas has traditionally been described in acute hydrops to provide tamponade to any break in the DM [11]. However, accuracy in the preparation of iso-expansile concentration is vital to prevent vision-threatening complications [12,13]. In a study by Siebelmann et al., Mi-OCT has been described as a useful tool for guided puncture and drainage of intrastromal fluid pockets with intracameral sulfur hexafluoride and pre-Descemetic sutures [14]. In the described case, Mi-OCT provided real-time feedback for the creation of a venting incision and helped to assess the decrease in fluid pocket intraoperatively. A surgical approach for venting incision was preferred along the inferotemporal quadrant to avoid subsequent scar formation along the visual axis. Compression sutures were avoided as venting incision was found to be sufficient for fluid drainage from the intrastromal cleft. Studies have described pre-Descemetic compression sutures as preferable over full-thickness corneal sutures to minimize endothelial damage and corneal scarring [15]. In cases of acute hydrops with DM scrolls and multiple clefts, full-thickness sutures are suitable for descemetopexy [16].

## Conclusions

Mi-OCT played a crucial role in managing a complex corneal hydrops case by providing real-time localization of stromal fluid clefts. Mi-OCT-guided venting incision, combined with intracameral gas, is a viable option for the rapid resolution of acute hydrops with a single large intrastromal fluid cleft. With appropriate management and favorable anatomy, hydrops resolution can occur as early as the first-day postprocedure.
